# High-Resolution TG-TOFMS Coupled with Principal Component Analysis and Kendrick Mass Defect Analysis: Elucidation of Molecular-Scale Degradation Behavior of Glass Fiber Reinforced Polypropylene during Thermo-Oxidative Degradation

**DOI:** 10.1021/acs.analchem.4c04630

**Published:** 2025-01-13

**Authors:** Taiki Ozawa, Sayaka Nakamura, Hiroaki Sato, Hideyuki Shinzawa, Hideaki Hagihara, Ryota Watanabe

**Affiliations:** Research Institute for Sustainable Chemistry, National Institute of Advanced Industrial Science and Technology (AIST), 1-1-1 Higashi, Tsukuba 305-8565, Japan

## Abstract

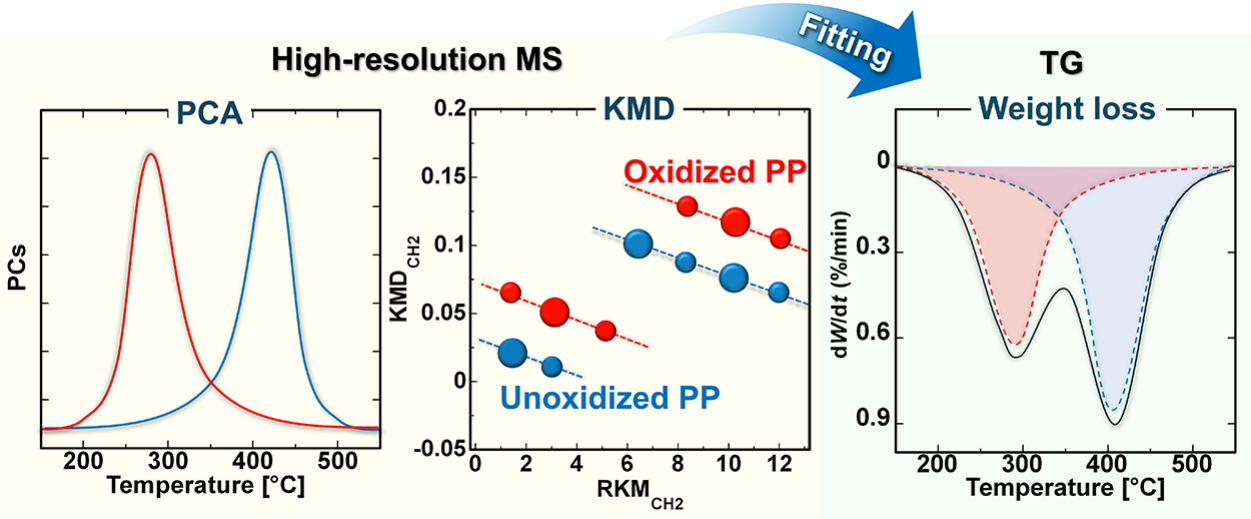

This study presents a novel approach that combines thermogravimetric analysis with time-of-flight mass spectrometry (TG-TOFMS), principal component analysis (PCA), and Kendrick mass defect (KMD) analysis—referred to as TG-PCA-KMD—to investigate molecular-scale structural changes and quantitatively assess the progression of thermo-oxidative degradation in glass fiber reinforced polypropylene (GF/PP). TG-TOFMS enables the simultaneous and sensitive detection of both structural changes due to thermo-oxidative degradation and compositional changes in the filler and matrix. PCA and KMD analysis are crucial for identifying specific ion series derived from the degraded PP matrix in the high-resolution mass spectra obtained through TG-TOFMS. Additionally, PCA fitting was employed to selectively extract information on the degraded components of GF/PP from differential thermogravimetric profiles. Our findings demonstrate the advantages and utility of TG-PCA-KMD in the degradation analysis of composite materials.

## Introduction

Glass fiber reinforced polypropylene (GF/PP) is widely used in structural components due to its high strength, thermal stability, low cost, and ease of recycling.^1−5^ Enhancing the durability of GF/PP against thermo-oxidative degradation is a critical concern, especially given the growing demand and production of GF/PP. Developing analytical techniques that can accurately capture molecular-scale structural changes in GF/PP during degradation is crucial for improving material durability based on the underlying degradation mechanisms.^2−7^

Thermo-oxidative degradation in GF/PP leads to the formation of oxidized polypropylene (PP) components.^8,9^ As degradation progresses, it causes scission of PP molecules and the formation of volatile components,^10^ resulting in a reduction of the PP matrix content in GF/PP. Therefore, to better understand the degradation mechanisms in GF/PP, it is essential to develop a quantitative analytical technique that can sensitively and simultaneously detect both the molecular-scale structural changes in the PP matrix due to oxidation and the compositional changes in the filler and matrix during the degradation process.

Evolved gas analysis-mass spectrometry using a pyrolyzer (EGA-MS) is an effective tool for determining the degradation structure of polymer molecules by analyzing the temperature-dependent mass spectra of products evolved during temperature ramping.^11−13^ However, EGA-MS is limited in that it cannot provide quantitative information on compositional changes, making it insufficient for analyzing the degradation of GF/PP.

Thermogravimetric analysis coupled with mass spectrometry (TG-MS) is recognized as an effective method for simultaneously observing both weight loss behavior and the mass spectra of polymer degradation products.^14−16^ Consequently, TG-MS is considered effective for quantitatively analyzing the degradation of GF/PP. However, when using TG-MS with a conventional quadrupole mass spectrometer, it is challenging to analyze the detailed structures of oxidation products due to insufficient mass resolution. For instance, distinguishing between ions such as C_3_H_7_^+^ (*m*/*z* 43.0542) and C_2_H_3_O^+^ (*m*/*z* 43.0178) in the mass spectrum is crucial for discussing the thermo-oxidative degradation of PP materials.^17^ Modern time-of-flight mass spectrometry overcomes this limitation by enabling the acquisition of high-resolution mass spectra, allowing these mass differences to be accurately distinguished.

Thus, thermogravimetric analysis coupled with time-of-flight mass spectrometry (TG-TOFMS) is a promising technique for precisely measuring the evolution of oxidized products from degradation during temperature ramping. However, interpreting the evolved products from temperature-dependent high-resolution mass spectra collected by TG-TOFMS is challenging due to the complexity of the data, which is often dominated by numerous peaks. To address this, we applied a data mining technique that combines principal component analysis (PCA) with Kendrick mass defect (KMD) analysis to temperature-dependent mass spectra.

PCA is a mathematical tool used to decompose two-way data into an orthogonal set of dominant factors known as eigenvectors.^18−21^ It generates two matrices—scores and loadings—which represent the features distributed throughout the data set. When applied to TG-TOFMS data, such as in the analysis of polymer samples before and after aging, PCA can play a crucial role in extracting key mass information about the specific evolved products resulting from degradation. Understanding the degradation behavior requires attributing specific mass data represented by PCA, although assigning numerous exact mass peaks can be extremely time-consuming. In this context, KMD analysis is invaluable. It categorizes and visualizes exact mass signals based on molecular compositions in a two-dimensional plot,^17,22−27^ facilitating the comprehensive attribution of ion series specific to degradation components.

In this study, we developed a technique called TG-PCA-KMD, which combines TG-TOFMS with PCA and KMD analyses. This approach enables simultaneous monitoring of the weight loss of polymer components and temperature-dependent high-resolution mass spectra. By applying this characterization technique, we elucidated the degradation mechanism of GF/PP by analyzing samples after accelerated aging at 180 °C under air exposure. The formation of oxidized products, identified through mass spectrometric analysis, was quantitatively interpreted in relation to the weight loss behavior observed in thermogravimetric (TG) analysis. Additionally, we developed a PCA fitting technique, which involves using a Gaussian fitting function on the shape of the PCA score plot. This was applied to differential thermogravimetric (DTG) curves to separate and individually evaluate the weight loss associated with the pyrolysis of oxidized products.

## Experimental Section

### Sample Preparation

Polypropylene (PP) from Prime Polymer (J-700GP, Japan), maleic anhydride grafted polypropylene (MAPP) from Kayaku Akzo (Kayabrid002PP, 2.0 wt % MA grafted, Japan), and glass fiber (GF) from Central Glass (ECS03–631 K, Japan) were used in this study. Before mixing, GF and all polymers were dried in an oven at 80 °C for 6 h. The GF/PP composite was fabricated using a twin-screw extruder (HK25D-41, Parker Corporation, Japan) equipped with a twin rotary mixer. PP and MAPP pellets, along with GF, were melt-mixed in a kneading machine at 180 °C and 150 rpm.

A sample sheet with a thickness of 0.5 mm was prepared from the melt-mixed material by hot pressing. The hot pressing process was conducted at 180 °C under 5 MPa for 3 min, followed by pressing under 10 MPa for 10 min using a hot-press machine (mini–Test Press-1, Toyo Seiki Seisakusho, Japan). The assembly included a Naflon sheet (Nichias, Japan), a stainless steel window frame (0.5 mm thick), and stainless steel plates. After hot pressing, the samples were slowly cooled to room temperature in the hot-press machine with the heating turned off. The final GF/PP weight ratio was PP:MAPP = 69:1:30.

### TG-TOFMS

The TG-TOFMS system used in this study comprised thermogravimetry (STA 2500 Regulus; NETZSCH, Germany), gas chromatography (7890 B; Agilent Technologies, USA), and time-of-flight mass spectrometry (JMST200, JEOL, Japan) equipped with an in-line evolved gas analysis (EGA) accessory (NETZSCH, Germany). For the TG-TOFMS measurements, a sample weight of approximately 1.0 mg was used, which was sufficiently small to ensure rapid thermodynamic equilibrium during programmed heating. The sample was placed in an aluminum sample cup (J1560180, NETZSCH, Germany) and heated from 100 to 550 °C at a rate of 10 °C per minute under a helium atmosphere.

A portion of the gas flow (70 mL/min) was continuously introduced into the mass spectrometer through a deactivated fused-silica column (10 m × 0.32 mm i.d., Agilent Technologies, USA), which was heated to 280 °C to prevent condensation of less volatile products in the capillary. Mass spectrometric measurements were performed using electron ionization (EI), with a mass range of *m*/*z* 30–800 and a recording interval of 0.5 s. The mass spectrometer was tuned using perfluorotributylamine (PFTBA), and peak resolution was adjusted to approximately 10,000 for *m*/*z* 501.970. The evolution profiles of the products were observed in total ion current (TIC) mode, which represents the sum of the intensities of all mass spectral peaks. The DTG data (d*W*/d*t*) were generated by differentiating the TG values and smoothing them using a moving average to reduce noise.

### Methods

#### PCA

In this paper, boldface capital letters represent matrices, and the superscript “t” denotes the transposition of a matrix. A series of exact mass data for the evolved products was collected using TG-TOFMS. The spectra can be represented as a matrix ***X*** with dimensions *m × n*, as expressed in eq 1.
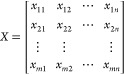
1where *m* is the number of spectra collected at different time intervals, and *n* is the number of data points along the *m*/*z* axis. By applying singular value decomposition (SVD), the matrix ***X*** representing the mass spectra can be expressed as the product of two matrices, ***T*** and ***P***, along with a residual matrix ***E***, as shown in eq 2:
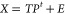
2

Here, ***T*** is the *m × r* PCA score matrix, and ***P*** is the *n × r* PCA loading matrix. The rank *r* represents the number of principal components (PCs) that capture a significant portion of the information within the mass spectral data ***X***. For instance, the first and second principal components, denoted as PC-1 and PC-2, respectively, are two key abstract components derived from the mass spectral series. The matrix ***E*** represents the residual portion of the original data that is not accounted for by the first *r* PCs. The matrix ***T*** contains abstract information on temperature-induced variations in the mass spectra of the evolved products, while ***P*** represents critical variables that provide chemically meaningful interpretations of the patterns observed in ***T***. A series of PCA were performed using MATLAB version R2024a and the Statistics and Machine Learning Toolbox (MathWorks, USA).

#### KMD Analysis

KMD analysis combined with high-resolutiKMD Analysison mass spectrometry, was conducted following the approach used in previous studies.^17,22^ KMD analysis is particularly effective in distinguishing between hydrocarbon ions and other structural features in a two-dimensional plot. The exact mass data were converted to Kendrick masses (KM) using eq 3.
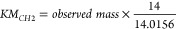
3

Kendrick mass (KM) when CH_2_ is used as the base unit is denoted as KM_CH2_. The unified atomic mass of CH_2_ is 14.0156, as defined by the International Union of Pure and Applied Chemistry (IUPAC). The KM_CH2_ value of each peak was rounded to obtain integer KM values, referred to as nominal KM_CH2_ (NKM_CH2_). The Kendrick mass defect value (KMD_CH2_), which is the difference between NKM_CH2_ and KM_CH2_, was calculated using eq 4.
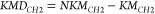
4

The NKM_CH2_ and KMD_CH2_ values are plotted on the *x*-axis and *y*-axis, respectively, in a bubble chart format to represent the distribution of each component. The remainders of Kendrick mass (RKM) plots were utilized to identify components consisting of hydrocarbons with different chemical structures from CH_2_. The RKM value is calculated using a modulo function, as expressed in eq 5.
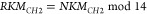
5where RKM_CH2_ is the RKM value obtained by using 14 (the integer mass of CH_2_) as the divisor. The RKM plot represents RKM as a function of NKM. A series of KMD analyses was conducted using MATLAB version R2024a (MathWorks, USA).

#### PCA Fitting

The Gaussian multipeak fitting method was employed to fit the temperature-dependent PCA score plots, utilizing no more than 10 Gaussian fitting peaks (Figure S1). All multipeak fittings aimed to minimize the chi-square values. Before PCA fitting, the score plots underwent linear baseline correction. The equation for Gaussian multipeak fitting is expressed in eq 6.^28,29^
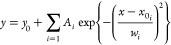
6where *i* = 0, 1, 2, 3, . . ., *y* is the peak intensity at *x*, *y*_*0*_ is the baseline, *A*_*i*_ is the peak intensity at *x*_*0i*_, *x*_*0i*_ is the peak center, and *w*_*i*_ is the full width at half-maximum. The Gaussian multipeak fitting functions generated from fitting PC-1 and PC-2 were utilized to fit the DTG curves. The equations for fitting PC-1 and PC-2 to the DTG curves are expressed in eq 7.
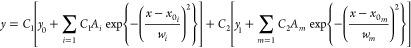
7where *C*_*1*_ and *C*_*2*_ are the constants used to fit PC-1 and PC-2 to the DTG curves, respectively. These fittings were conducted using Igor Pro 9 version 9.05 (Wavemetrics, USA).

#### TG-PCA-KMD

**Figure 1:**
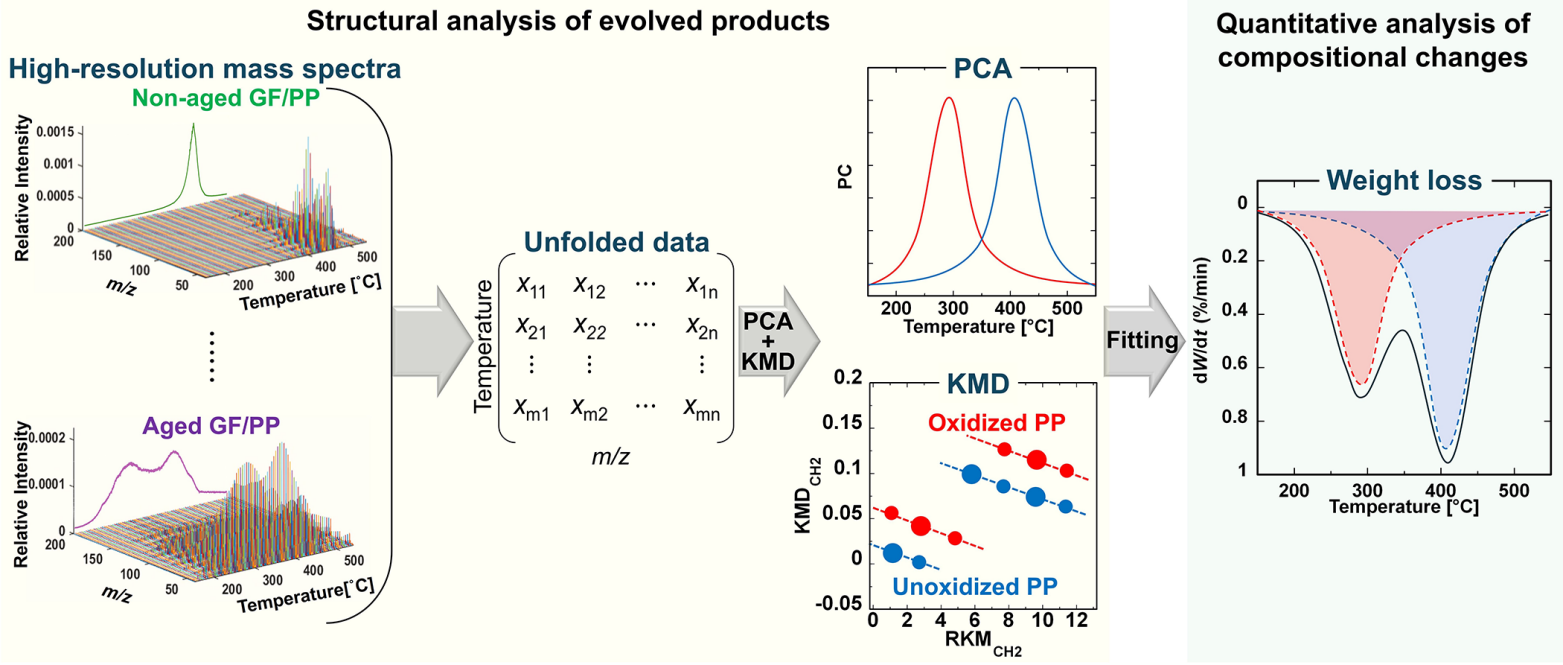
A schematic illustration of the detailed mechanisms of GF/PP degradation as elucidated by the TG-PCA-KMD approach.

Figure 1 presents a schematic illustration of the TG-PCA-KMD approach for elucidating changes in the structure and composition of GF/PP upon aging. TG-TOFMS measurements were performed on both original and aged samples, yielding many high-resolution mass spectra from a single measurement. Feature extraction using PCA effectively identifies ion series specific to the products evolved from the degraded PP components amid such vast data. PCA was applied to the unfolding results in a two-way data array, which was converted from three-dimensional data (mass-to-charge ratio (*m*/*z*), temperature, and aging time) to generate the Score ***T*** and Load ***P*** matrices. The spectral changes induced by aging treatment in the polymer system can be elucidated by examining the patterns observed in the plots of these scores against the ramping temperature. The loadings of PC-1 and PC-2 represent the exact mass signals of the data set used in PCA, providing chemical interpretations of the patterns observed in the PCA scores. KMD analysis was employed to comprehensively analyze the molecular formulas of the characteristic ions extracted by the PCA loadings. The evolved products resulting from the degradation of GF/PP were identified through KMD analysis. Furthermore, the degradation mechanism of GF/PP was validated by correlating the quantitative analysis of compositional changes, obtained via PCA fitting of the DTG curves, with the structural analysis results of the evolved products.

## Results and Discussion

### TG-TOFMS Analysis

**Figure 2:**
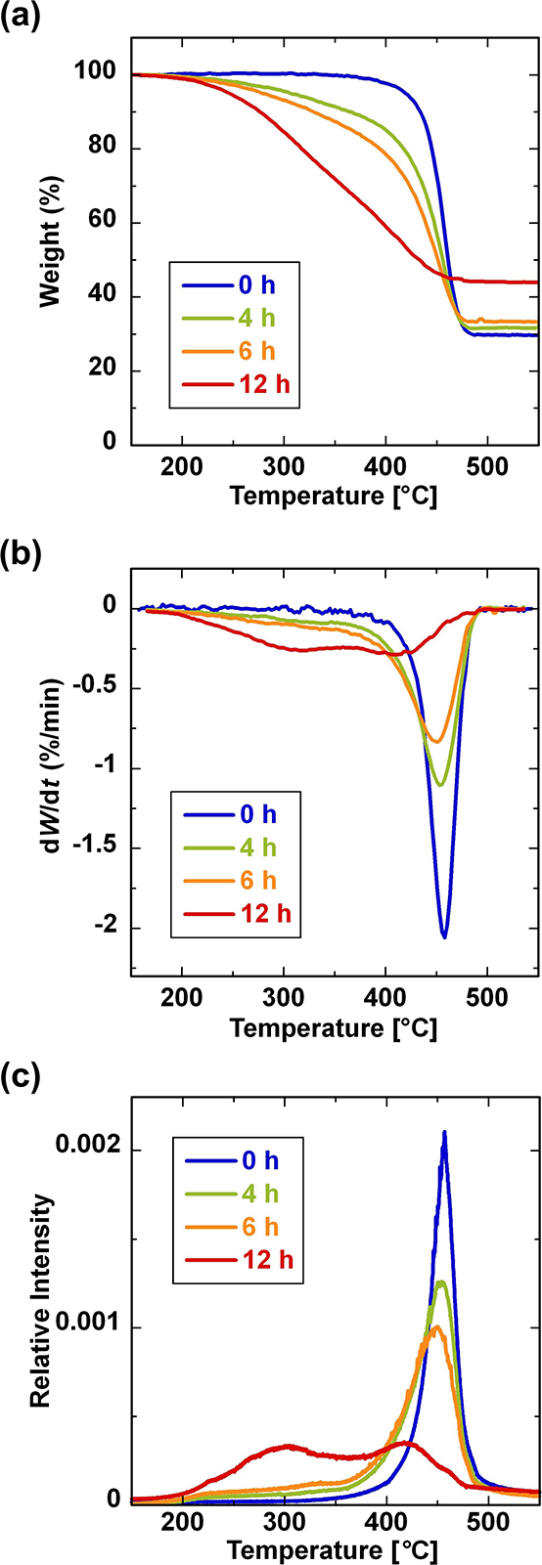
TG-TOFMS analysis of GF/PP samples aged at 180 °C for 0, 4, 6, and 12 h: (a) TG curves, (b) DTG curves, and (c) evolution profiles of the products using TOFMS in TIC mode.

TG-TOFMS analysis was conducted on GF/PP samples aged at 180 °C for 0, 4, 6, and 12 h to elucidate the structural changes occurring during the aging process (Figure 2). The aging times were determined based on isothermal *in situ* Fourier transform infrared spectroscopy (FTIR) measurements (Figure S2). Figure 2a presents the TG curves for GF/PP aged at 180 °C for the specified durations. The weight losses observed in all samples were primarily attributed to the pyrolysis of the polymer matrix. The original GF/PP without aging treatment exhibited rapid weight loss in the temperature range of 400–480 °C. Notably, the weight-loss temperature decreased with increasing aging time, indicating that the thermal stability of the PP matrix diminished as degradation progressed. Changes in the TG curves were further illustrated in the corresponding DTG curves (Figure 2b). The residue amount of the original GF/PP after heating to 550 °C was found to be 29.7%, consistent with the preparation ratio of GF (Figure 2a). This suggests that the PP matrix was completely decomposed at 550 °C, leaving only the GF as the residue. Additionally, no discoloration was observed in the residue, indicating the absence of carbonized components, even in the aged samples (Figure S3). Therefore, the amount of residue accurately reflected the filler ratio in the GF/PP samples. As aging time increased, the residue amount also increased, suggesting the proportion of PP matrix reduced during the aging process.

TG-TOFMS simultaneously captured temperature-dependent mass spectra during the TG measurements. Figure 2c illustrates the evolution profiles of the products from GF/PP aged for 0, 4, 6, and 12 h during the TG process, analyzed in TIC mode. These profiles were constructed by summing the intensities of the mass peaks at *m*/*z* 30–800 in the mass spectra. The TIC curves were normalized by the total peak area of all mass peaks collected during heating from 150 to 550 °C. The evolution patterns of the products closely matched the weight loss behavior observed in the TG analysis. The changes in weight loss and the evolution profiles of the products from the aged samples can be attributed to the structural changes in GF/PP due to aging. However, the detailed structural changes in GF/PP during aging remain unclear. Interpreting the temperature-dependent mass spectra can enhance our understanding of the structural changes in GF/PP resulting from the aging treatment.

**Figure 3:**
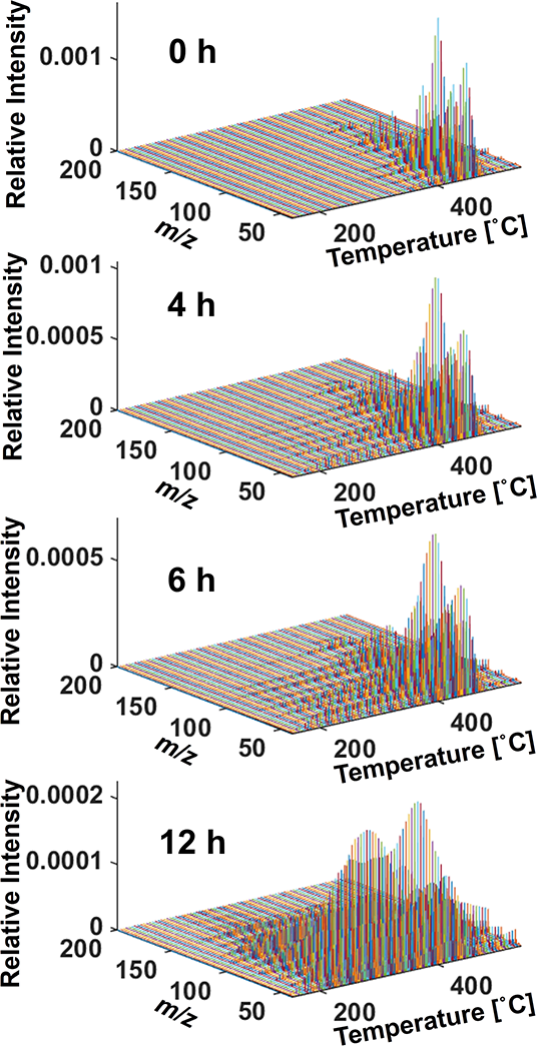
Temperature-dependent mass spectra of evolved products from GF/PP samples aged at 180 °C for 0, 4, 6, and 12 h.

The temperature-dependent mass spectra of evolved products from GF/PP samples aged for 0, 4, 6, and 12 h were collected across the temperature range of 150–550 °C (Figure 3). These spectra were obtained by summing the mass spectra acquired at 5 °C intervals. Several mass spectra of the pyrolysis products from the PP matrix in the GF/PP samples were observed. However, analyzing the intensity variations of specific exact mass peaks can be labor-intensive. PCA is a valuable technique for extracting subtle yet relevant information from the extensive high-resolution mass spectra.

### PCA

**Figure 4:**
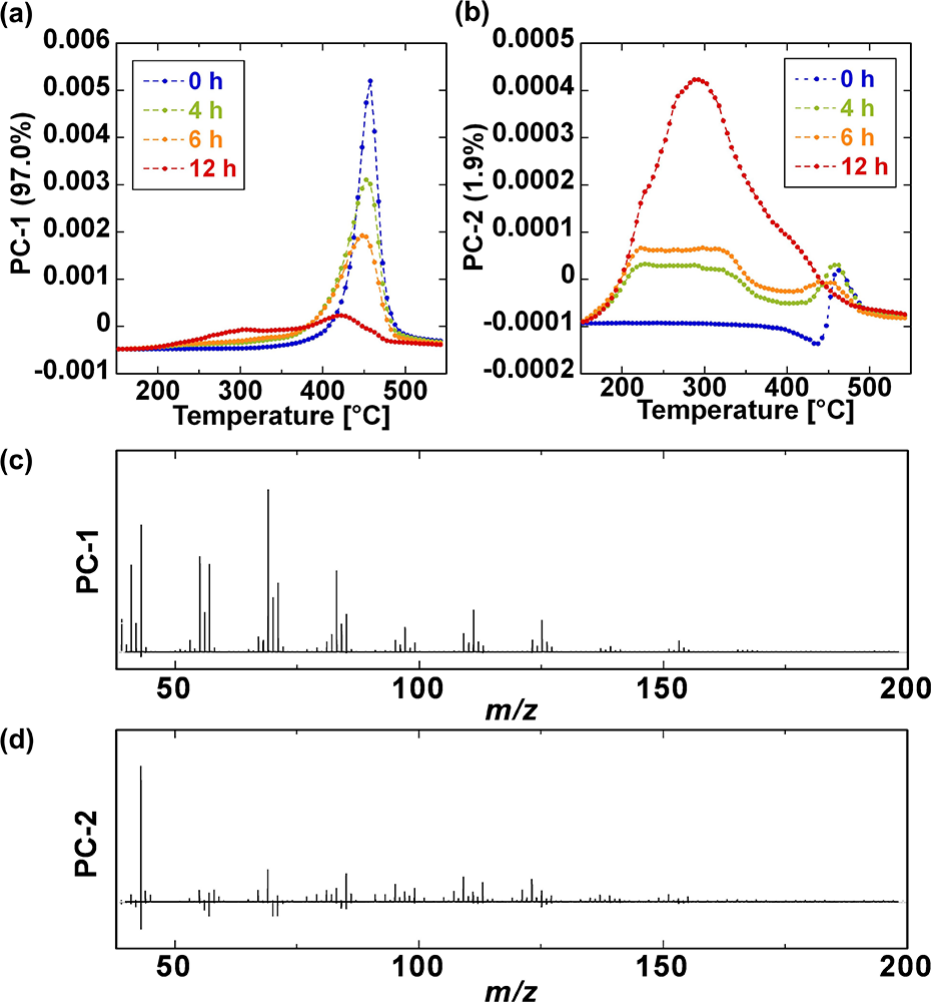
Temperature-dependent score plots of (a) PC-1 and (b) PC-2, along with the corresponding loading plots of (c) PC-1 and (d) PC-2. PCA was applied to the temperature-dependent mass spectra of GF/PP samples with varying aging times.

To identify the specific ion series arising from the evolved products of degraded GF/PP, PCA was performed simultaneously on the entire data set of temperature-dependent mass spectra from the GF/PP samples aged for 0, 4, 6, and 12 h (Figure 4). The first principal component (PC-1) and the second principal component (PC-2) accounted for 97.0% and 1.9% of the variance in the analyzed spectra, respectively. The temperature-dependent scores for PC-1 are illustrated in Figure 4a. In the temperature range of 400–480 °C, the PC-1 scores decreased with increasing aging time, while scores at approximately 300 °C increased. In contrast, the variation in PC-2 scores exhibited a markedly different trend. The PC-2 scores in the temperature range of 200–400 °C consistently increased as aging time progressed (Figure 4b). This suggests that the ions contributing to the positive direction of PC-2 variation are related to products evolved from the degraded components.

The loading plots provided chemically and physically meaningful interpretations of the patterns observed in the score plots. Figures 4c and 4d present the corresponding loading plots for PC-1 and PC-2, respectively. The PC-1 loading plot exhibited only positive peaks across the *m*/*z* region, which were associated with ion series that either monotonically increased or decreased with evolving temperature. Conversely, the PC-2 loading plot contained both positive and negative peaks, contributing to the positive and negative values of the PC-2 scores, respectively. As shown in Figure 4b, the PC-2 scores shifted toward the positive direction with increasing aging time, indicating that attention should be focused on the positive peaks in the PC-2 loading to elucidate the degradation structures of the PP matrix.

However, the extraction of numerous peaks from the loading plots, constructed from exact mass data, made it challenging to comprehensively assign each peak. Therefore, KMD analysis was applied as an effective data mining technique for attributing specific ions indicated by the PC-1 and PC-2 loadings.

### KMD Analysis

**Figure 5:**
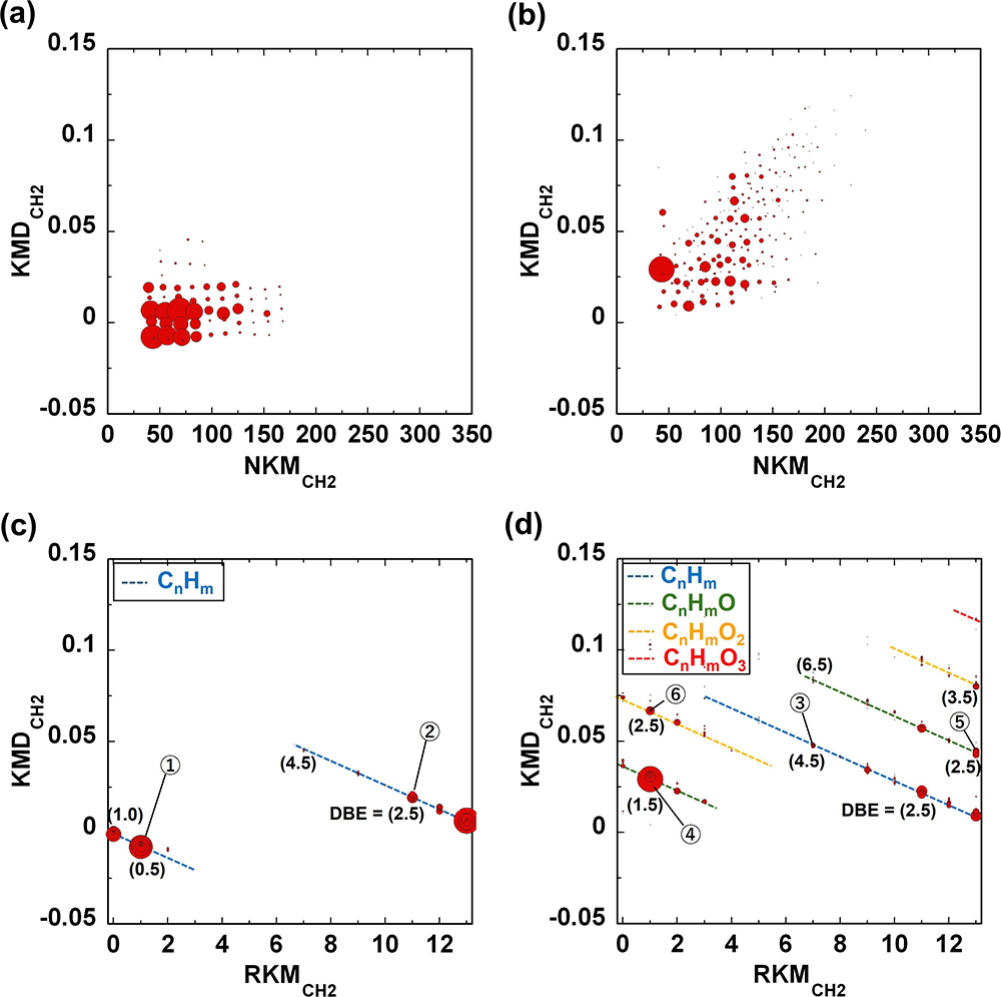
KMD and RKM plots of PCA loadings. KMD plots of positive peaks for (a) PC-1 and (b) PC-2. RKM plots of positive peaks for (c) PC-1 and (d) PC-2. The dashed lines in the RKM plots represent different classes of hydrocarbons: blue for hydrocarbons, green for hydrocarbons containing one oxygen atom, yellow for hydrocarbons containing two oxygen atoms, and red for hydrocarbons containing three oxygen atoms. The numbers in parentheses indicate the double-bond equivalents (DBE).

The positive peaks from the PC-1 and PC-2 loadings were converted into KMD plots by using CH_2_ as the base unit (Figure 5). In these KMD plots, the area of each dot correlates with the number of evolved ions. In the KMD plots of the positive peaks from the PC-1 loading, the distribution of hydrocarbon ions is predominantly represented in a band shape with KMD_CH2_ values of ±0.02 (Figure 5a). In contrast, the KMD plot of the positive peaks from the PC-2 loading shows additional dots distributed at KMD_CH2_ values greater than 0.02, which correspond to ion series specific to the evolved products from the degraded components (Figure 5b). However, the overlapping distributions in the plots make it challenging to clearly distinguish individual ions.

To address this, the KMD plots of the positive peaks for PC-1 and PC-2 were further converted into RKM plots, as shown in Figures 5c and 5d, respectively. The RKM plots facilitate the compression and summarization of the carbon number distribution data, highlighting differences in chemical structures, such as functional groups and degrees of unsaturation. In the RKM plots, ions with different double bond equivalents (DBE)—which indicate the degree of unsaturation—are arranged diagonally, as represented by the dashed lines. The DBE value was calculated using the molecular formula C_c_H_h_O_o_, as given in eq 8.
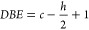
8

The RKM plots of the positive peaks from the PC-1 loading indicate that the PC-1 components are associated with hydrocarbon ions lacking heteroatoms, displaying a DBE range of 0.5 to 4.5 (Figure 5c). This suggests that PC-1 is derived from an ion series that evolved from the unoxidized domain of the PP matrix. Furthermore, the KMD and RKM plots of PC-1 closely resemble those of the pyrolysis products from the untreated original PP (Figure S4). This strongly supports the correctness of the attribution of PC-1. In contrast, the RKM plots of the positive peaks from the PC-2 loading reveal oxidized hydrocarbon ions containing one to three oxygen atoms, with varying DBE values (Figure 5d). Furthermore, hydrocarbons with a DBE range of 1.5 to 7.0 are present in this RKM plot of PC-2 loading. The presence of these unsaturated compounds suggests that they were likely generated by the detachment of oxidizing functional groups from the degraded PP molecules during pyrolysis. The KMD analysis of the slight negative peaks observed in the PC-2 loading plot represents simple hydrocarbon compounds without heteroatoms, likely originating from unoxidized PP (Figure S5). However, the influence of this component on variations in the PC-2 score is limited.

A series of spectral analyses revealed that the PC-1 and PC-2 components were derived from the pyrolysis products of unoxidized and oxidized PP, respectively. The representative ions selected from the ion series labeled (①–⑥) in Figures 5c and 5d are summarized in Table 1. The attributes of each ion were determined through compositional analysis based on their exact mass values. Ion ① is a hydrocarbon ion extracted from the PC-1 loading, originating from unoxidized PP, with a DBE of 0.5. Ions ② and ③, extracted from both PC-1 and PC-2 loadings, are hydrocarbon ions with higher DBE values than ion ①, indicating they are generated from both unoxidized and oxidized PP. Ions ④,⑤, and ⑥ are oxidized hydrocarbon ions derived from the positive peaks of the PC-2 loading, originating from oxidized PP. Notably, ions ⑤ and ⑥ contain additional oxygen atoms compared to ion ②.

**Table 1:** Assigned Structures of Representative Peaks Observed in the Loading Plots of PC-1 and PC-2

Entry	*m*/*z*	Molecular formular	DBE	Source
①	43.0557	C_3_H_7_^+^	0.5	Unoxidized PP
②	81.0707	C_6_H_9_^+^	2.5	Unoxidized and oxidized PP
③	77.0403	C_6_H_5_^+^	4.5	Unoxidized and oxidized PP
④	43.0187	C_2_H_3_O^+^	1.5	Oxidized PP
⑤	97.0633	C_6_H_9_O^+^	2.5	Oxidized PP
⑥	113.0591	C_6_H_9_O_2_^+^	2.5	Oxidized PP

The KMD analysis facilitates the immediate and precise attribution of specific ion series based on the PCA output. The detailed evolution behavior of ions ①–⑥ was examined using the extracted ion monitoring (EIM) mode of the TG-TOFMS system, providing deeper insights into the thermo-oxidative degradation of the GF/PP composite (Figure S6). The shape of the score plot corresponds to the pyrolysis behavior of each PP component. Consequently, we investigated the compositional changes during aging using PCA fitting of the DTG curves.

### PCA Fitting

**Figure 6:**
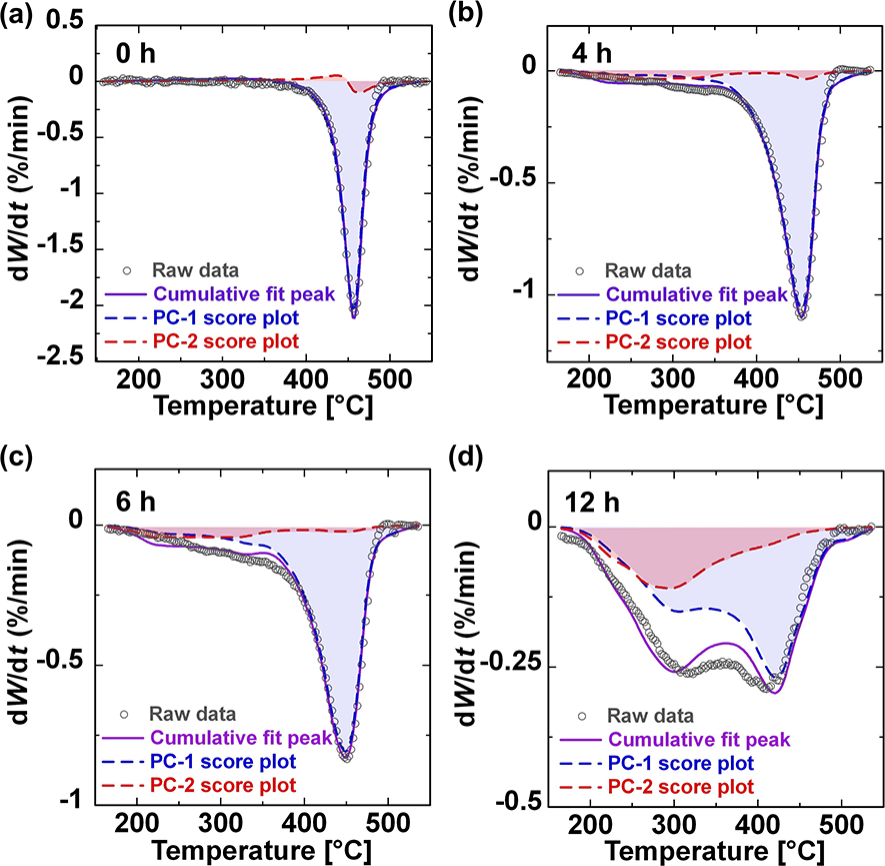
Fitting of PC-1 and PC-2 to the DTG curves of GF/PP aged for (a) 0, (b) 4, (c) 6, and (d) 12 h. The circles represent the raw DTG data, while the purple lines indicate the cumulative fitted peaks. The blue and red dashed lines represent the fitted peaks for PC-1 and PC-2, respectively.

The component ratios of unoxidized and oxidized PP were calculated using PCA fitting of the DTG curves for GF/PP aged at 180 °C for 0, 4, 6, and 12 h (Figure 6). Figure 6a presents the PCA fitting results for the original GF/PP without aging treatment, showing that the fitting area for the PC-1 score is significantly larger than that for the PC-2 score. In Figures 6b–d, the PCA fitting results for the aged GF/PP demonstrate a clear increase in the area ratio of PC-2 to PC-1. This PCA fitting technique enables the separation and individual evaluation of weight loss associated with the principal components extracted from the high-resolution mass spectra.

**Figure 7:**
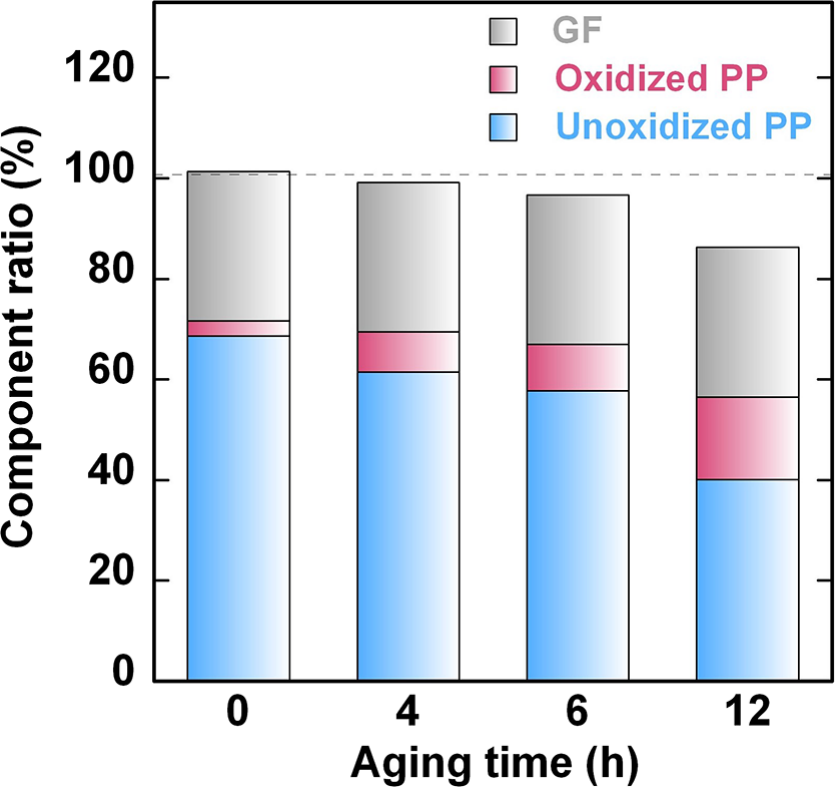
Changes in the weight ratios of unoxidized PP (blue) and oxidized PP (red) with increasing aging time. The weight ratio of GF remains constant at 29.7%. The decrease in the total component ratio indicates the amount of gasified PP.

The component ratios of unoxidized and oxidized PP were determined from the fitting areas of the PC-1 and PC-2 score plots to the DTG curves of the GF/PP samples (Figure 7). The untreated GF/PP comprised 68.7% unoxidized PP, 2.9% oxidized PP, and 29.7% GF, as estimated by combining the PCA fitting results with the residue content. The proportion of unoxidized PP decreased to 40.0% after 12 h of aging. In contrast, the ratio of oxidized PP consistently increased to 16.5% after the same aging period.

Considering the total component ratio of GF/PP samples—including the GF content, which remains unchanged during the aging treatment at 180 °C—the reduction in the total component ratio can be interpreted as the loss of gasified PP. This loss is attributed to chain scission caused by further oxidative degradation. Specifically, after aging for 4, 6, and 12 h, 0.8%, 3.3%, and 13.7% of the GF/PP components were lost, respectively, due to the gasification of the PP matrix. The total amount of degraded PP was the sum of oxidized and gasified PP during the aging process, indicating that 8.9%, 12.5%, and 30.2% of PP was degraded from the initial 70.3% of PP in GF/PP after aging for 4, 6, and 12 h, respectively. This quantitatively demonstrates that the amount of degraded PP components in GF/PP increased with extended aging time.

The collective findings of this study suggest that the TG-PCA-KMD technique is valuable for revealing the detailed structure of thermo-oxidized PP and providing quantitative insights into the structural changes in GF/PP during the aging process. This insight significantly enhances our comprehension of the degradation mechanisms of composite materials.

## Conclusions

The TG-PCA-KMD technique was developed as an innovative characterization method to interpret the structural changes in GF/PP during thermo-oxidative aging, leading to a deeper understanding of the degradation mechanisms of this composite material. The pyrolysis temperature of the PP matrix decreased with increasing aging time, indicating a loss of thermal stability. This study aimed to analyze the temperature-dependent mass spectra collected using TG-TOFMS, providing meaningful interpretations of the pyrolysis behavior of GF/PP throughout the aging process. PCA and KMD analysis were performed on the temperature-dependent mass spectra of GF/PP at various aging times to identify specific ion series arising from oxidation products. The evolution behaviors of unoxidized and oxidized PP corresponded to the scores of PC-1 and PC-2, respectively. The total ratio of degraded PP in GF/PP was determined by summing the oxidized PP estimated from PCA fitting of the DTG curves and the gasified PP estimated from the reduction in the total component ratio. The total ratio of degraded PP in GF/PP increased significantly from 2.9% to 30.2% after 12 h of aging.

The integrated approach of TG-PCA-KMD provides a comprehensive understanding of the degradation mechanism of GF/PP by detailing the structural changes during thermo-oxidative degradation. Furthermore, TG-PCA-KMD facilitated a quantitative evaluation of degradation states based on the weight loss of the degraded components. This study highlights the effectiveness of TG-PCA-KMD in elucidating degradation mechanisms and quantitatively interpreting degradation in polymer composites like GF/PP.

## Supporting Information Available

Details on the Gaussian multipeak fitting method used to fit the PCA score plots (Figure S1), isothermal *in situ* FTIR measurements (Figure S2), the residue of the GF/PP sample after heating to 550 °C (Figure S3), KMD analysis of the pyrolysis products of original PP (Figure S4), KMD analysis of negative peaks for PC-2 (Figure S5), and the evolution behavior of representative ions observed through the Extracted Ion Monitoring (EIM) mode (Figure S6) (PDF)
