# Supplementary material for: High-Resolution TG-TOFMS Coupled with Principal Component Analysis and Kendrick Mass Defect Analysis: Elucidation of Molecular-Scale Degradation Behavior of Glass Fiber Reinforced Polypropylene during Thermo-Oxidative Degradation
Source: Anal Chem. 2025 Jan 13;97(3):1665–72. doi: 10.1021/acs.analchem.4c04630 (PMC11780572; doi:10.1021/acs.analchem.4c04630)
Supplement: Supplementary file 1 — ac4c04630_si_001.pdf [file ac4c04630_si_001.pdf]

Supporting information

High-Resolution TG-TOFMS Coupled with Principal Component Analysis and Kendrick Mass Defect Analysis: Elucidation of Molecular-Scale Degradation Behavior of Glass Fiber Reinforced Polypropylene during Thermo-Oxidative Degradation

Taiki Ozawa, Sayaka Nakamura, Hiroaki Sato, Hideyuki Shinzawa, Hideaki Hagihara, and Ryota Watanabe*

Research Institute for Sustainable Chemistry, National Institute of Advanced Industrial Science and Technology (AIST), 1-1-1 Higashi, Tsukuba 305-8565, Japan

Tel: +81-50-3522-3901

Correspondence author.

E-mail address: r.watanabe@aist.go.jp (R. Watanabe)

TABLE OF CONTENTS

1. Gaussian Multipeak Fitting Method for PCA Score Plots ········· S3

2. Isothermal *In-Situ* FTIR ········· S6

3. Residue of GF/PP Sample after Heating to 550 ˚C ········· S8

4. KMD Analysis of Pyrolysis Products of Original PP ········· S8

5. KMD Analysis of Negative Peaks for PC-2 ········· S9

6. Evolution Behavior of Representative Ions Observed by EIM Mode ········· S10

1. Gaussian Multipeak Fitting Method for PCA Score Plots

The Gaussian multipeak fitting method was employed to fit the PCA score plots in relation to the DTG curves (**Figure S1**). The chi-square value (χ^2) indicates the goodness of fit, with all multipeak fittings performed to minimize this value. Baseline correction was applied to ensure that the PCA score was zero at 150 and 550 ˚C.

- **Figure S1a** shows the multipeak fitting results of PC-1 for the original GF/PP, using the baseline and nine Gaussian peaks.
- **Figure S1b** shows the multipeak fitting results for PC-2 of the original GF/PP, using the baseline and seven Gaussian peaks.
- **Figure S1c** shows the multipeak fitting results for PC-1 of GF/PP aged at 180 ˚C for 4 h, using the baseline and nine Gaussian peaks.
- **Figure S1d** shows the multipeak fitting results for PC-2 of GF/PP aged at 180 ˚C for 4 h, using the baseline and nine Gaussian peaks.
- **Figure S1e** shows the multipeak fitting results for PC-1 of GF/PP aged at 180 ˚C for 6 h, using the baseline and eight Gaussian peaks.
- **Figure S1f** shows the multipeak fitting results for PC-2 of GF/PP aged at 180 ˚C for 6 h, using the baseline and ten Gaussian peaks.
- **Figure S1g** shows the multipeak fitting results for PC-1 of GF/PP aged at 180 ˚C for 12 h, using the baseline and six Gaussian peaks.
- **Figure S1h** shows the multipeak fitting results for PC-2 of GF/PP aged at 180 ˚C for 12 h, using the baseline and five Gaussian peaks.

The peaks of the cumulative fit overlapped with the score plots of PC-1 and PC-2 with high accuracy, and the chi-square values were sufficiently small, indicating successful fitting. All fittings were conducted using the Multipeak Fit feature of Igor Pro 9 (version 9.05; Wavemetrics, USA).

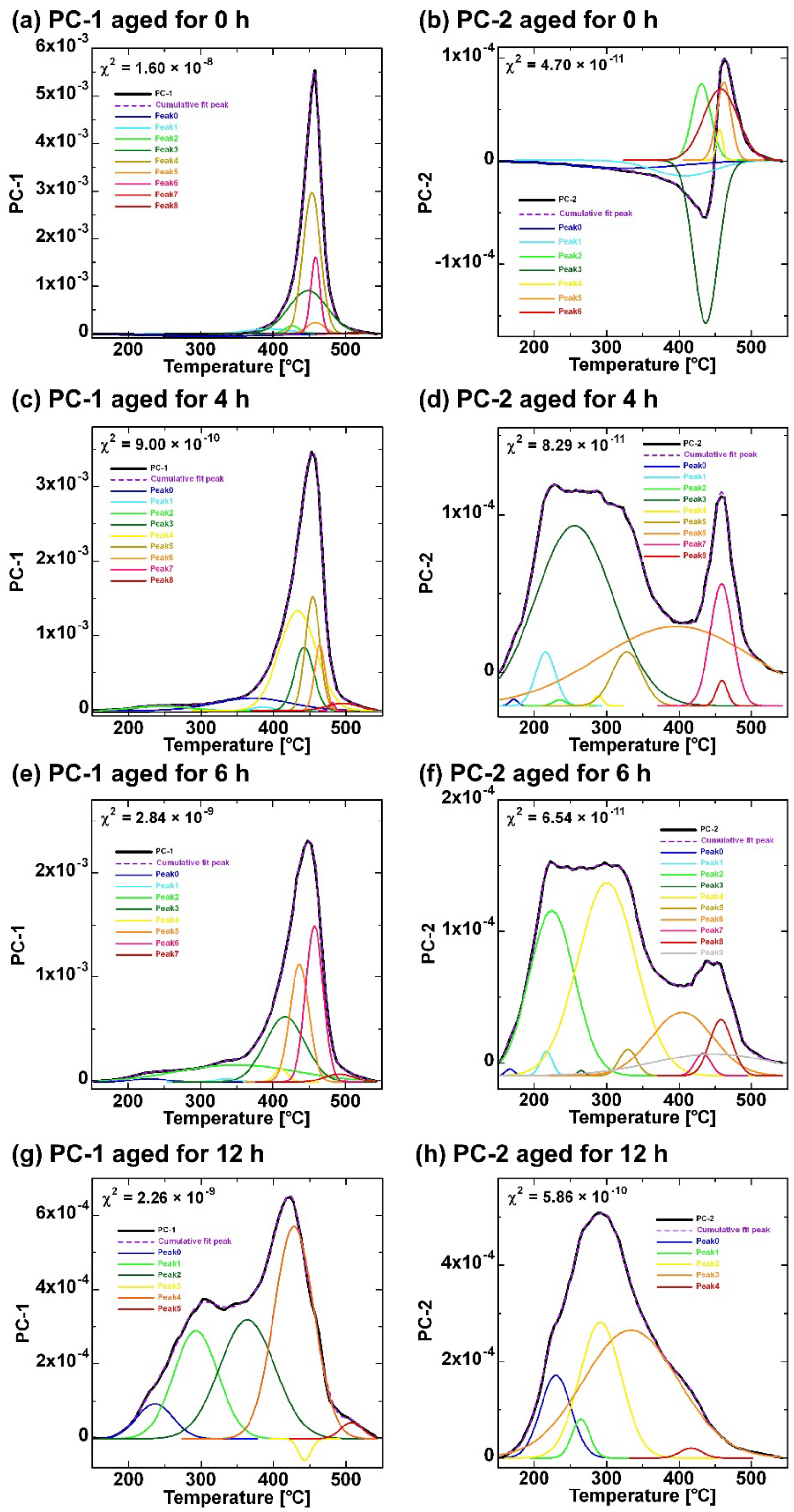

Figure S1. Gaussian multipeak fitting results of temperature-dependent PCA score plots constructed from GF/PP aged for (a,b) 0, (c,d) 4, (e,f) 6, and (g,h) 12 h. The PC-1 scores for GF/PP aged for (a) 0, (c) 4, (e) 6, and (g) 12 h are shown in the left column, while the PC-2 scores for the same aging times are displayed in the right column (b, d, f, h). The χ^2 value indicates the goodness of fit. The black line represents the raw PC-1 and PC-2 score plots, the purple lines indicate the cumulative fitted peaks, and the other lines correspond to the individual Gaussian peaks.

2. Isothermal *In-Situ* FTIR

In-situ FTIR measurements were conducted following a previously described method[1] using a Nicolet 6700 FTIR spectrometer (Thermo Scientific, USA) coupled with a GladiATR (PIKE Technologies, USA) equipped with a diamond-attenuated total reflection (ATR) prism. This setup was utilized to monitor the thermo-oxidative degradation state. The FTIR spectra were averaged over 256 scans with a resolution of 4 cm^{-1}. A sample was placed on the ATR prism, which was heated to 180 ˚C in an air atmosphere. Spectra were collected at 10 min intervals. Data acquisition was performed using TempPRO 7 software (PIKE Technologies, USA), and the data were subsequently imported into MATLAB version R2024a (MathWorks, USA). The spectra were pretreated using Savitzky–Golay smoothing with 11 points and subjected to a baseline offset.

To investigate the behavior and time scale of thermo-oxidative degradation of GF/PP, isothermal *in-situ* FTIR measurements were performed at 180 ˚C (**Figure S2**). A thin sample film with a thickness of 20 μm was utilized for the FTIR measurements. This technique is capable of probing structural changes within the first 20 μm near the sample surface[1]. **Figure S2a** displays the time-dependent FTIR spectra of GF/PP during the aging process at 180 ˚C, specifically in the 1850–750 cm^{-1} region. With prolonged aging, absorption bands in the 1850–1650 cm^{-1} and 1300–900 cm^{-1} regions emerged. The band at 1850–1650 cm^{-1} results from a combination of several C=O stretching bands, including those from ketones at 1718 cm^{-1}, aldehydes at 1734 cm^{-1}, and peresters at 1765 cm^{-1}.[2] The band in the 1300–900 cm^{-1} range is attributed to CO stretching and OH bending modes. The increase in the intensities of these bands suggests the formation of oxidized PP during the aging process. Conversely, the absorption intensities of the CH_3 groups, specifically the asymmetric bending at 1458 cm^{-1} and

the symmetric bending at 1377 cm^{-1}, decreased with longer aging times.[3,4] These spectral changes indicate the thermo-oxidative degradation of the PP component.

To further explore the aging behavior, the carbonyl index was calculated from the time-dependent FTIR spectra (**Figure S2b**). The carbonyl index is defined as the ratio of the integrated absorption intensities of the C=O groups (1850 to 1650 cm^{-1}) to those of the CH_3 groups (1500 to 1420 cm^{-1}).[5] After 2 h of aging, the carbonyl index began to rise and reached its maximum after 12 h. Based on the carbonyl index, TG-TOFMS analysis was performed on the GF/PP samples aged at 180 ˚C for 0, 4, 6, and 12 h.

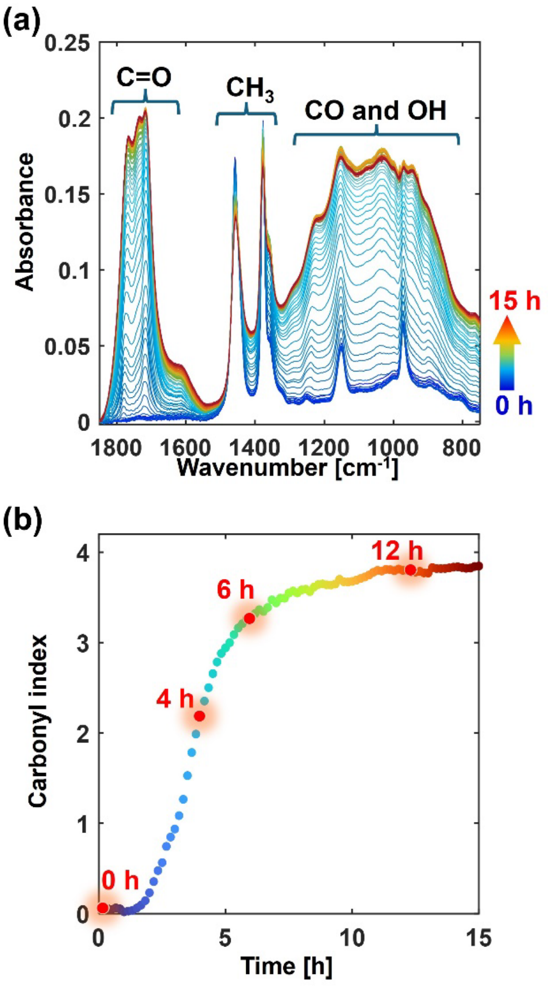

Figure S2. Changes in the FTIR spectra of GF/PP during the aging process at 180 ˚C. (a) Time-dependent FTIR spectra of GF/PP. (b) Carbonyl index calculated as the ratio of the integrated absorption intensities of the C=O groups (1850 to 1650 cm^{-1}) to the CH_3 groups (1500 to 1420 cm^{-1}) obtained from the time-dependent FTIR spectra.

3. Residue of GF/PP Sample after Heating to 550 ˚C

Figure S3 presents an image of a GF/PP sample that was aged at 180 ˚C for 12 h and subsequently heated to 550 ˚C. No discoloration was observed in the residue, indicating the absence of carbonized components. Additionally, the presence of white fibrous materials suggests the presence of glass fibers.

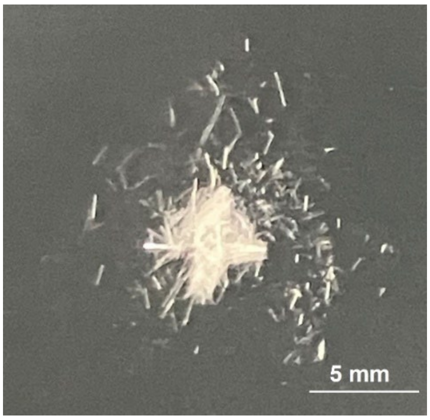

Figure S3. Image of the GF/PP sample aged at 180 ˚C for 12 h following the TG-TOFMS measurement.

4. KMD Analysis of Pyrolysis Products of Original PP

Figure S4 presents KMD and RKM plots of pyrolysis products of untreated original PP. In the KMD plot of pyrolysis products of original PP, the distribution of hydrocarbon ions is predominantly represented in a band shape with KMD_{CH2} values of ±0.02 (**Figure S4a**). The RKM plots of pyrolysis products of original PP indicate that the pyrolysis products of original PP are associated with hydrocarbon ions lacking heteroatoms, displaying a double-bond equivalents (DBE) range of 0 to 4.5 (**Figure S4b**).

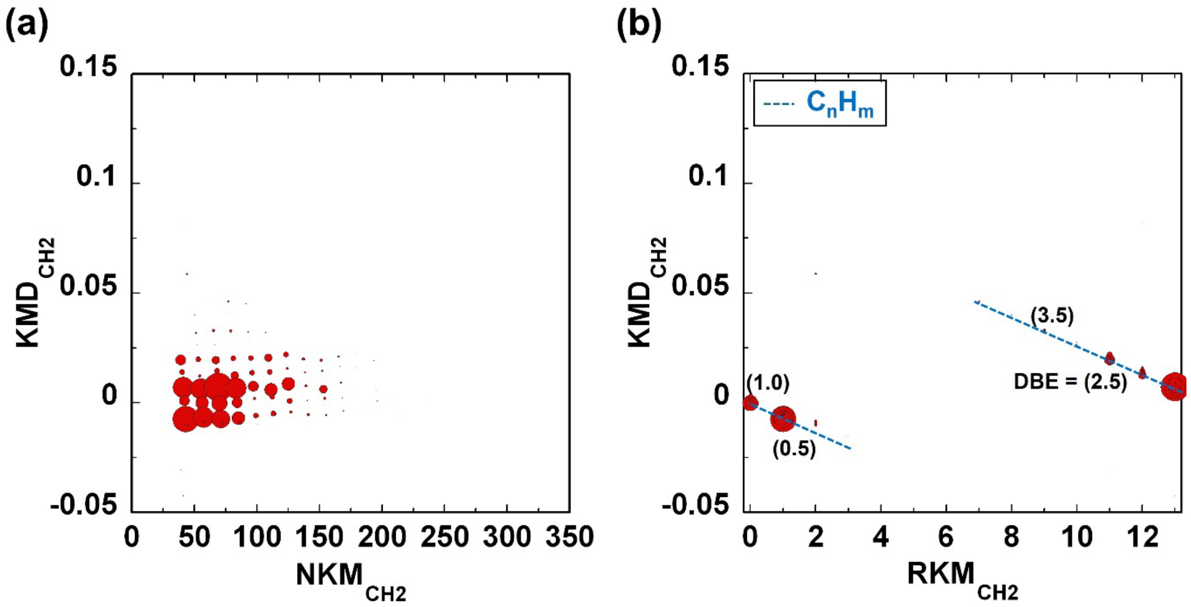

Figure S4. KMD analysis of pyrolysis products of original PP without GF; (a) KMD and (b) RKM plots. The dashed lines in the RKM plots represent hydrocarbons. The numbers in parentheses indicate the double-bond equivalents (DBE).

5. KMD Analysis of Negative Peaks for PC-2

Figure S5 presents KMD and RKM plots of negative peaks for PC-2. In the KMD plot of the negative peaks from the PC-2 loading, the distribution of hydrocarbon ions is predominantly represented in a band shape with KMD_{CH2} values of ±0.02 (**Figure S5a**). The RKM plots of the negative peaks from the PC-2 indicate that the PC-2 components are associated with hydrocarbon ions lacking heteroatoms, displaying a DBE range of 0 to 2.5 (**Figure S5b**).

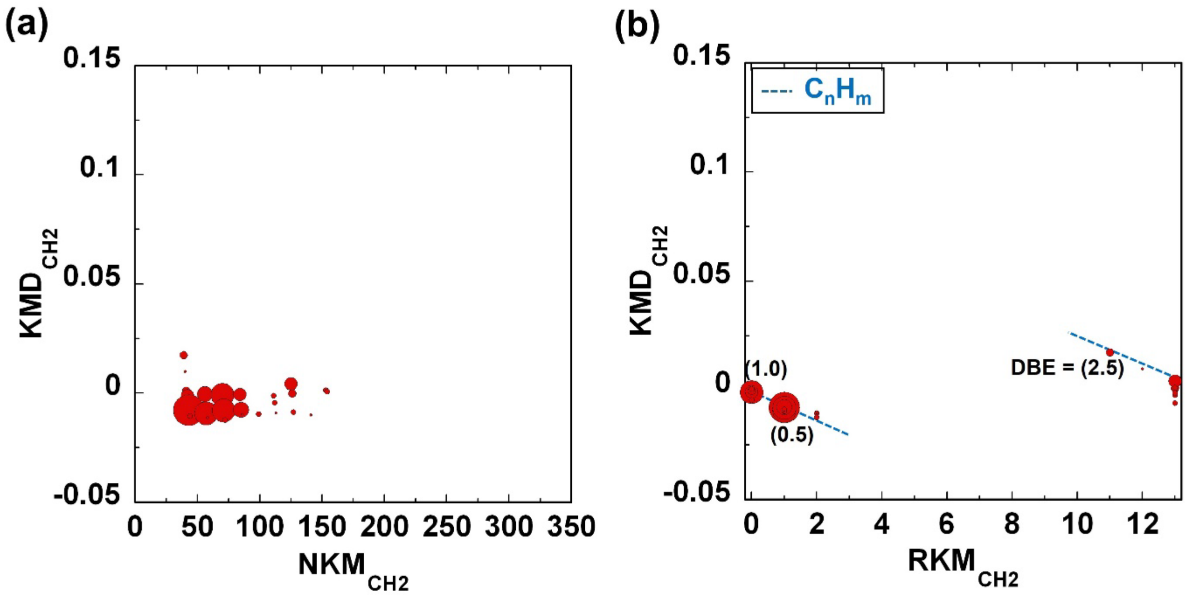

Figure S5. KMD analyses of negative peaks from PC-2 loading; (a) KMD and (b) RKM plots. The dashed lines in the RKM plots represent hydrocarbons. The numbers in parentheses indicate the DBE.

6. Evolution Behavior of Representative Ions Observed by EIM Mode

The detailed evolution behavior of the ions ①−⑥ listed in **Table 1.** in the manuscript was observed using the EIM mode of the TG-TOFMS system, providing deeper insight into the thermo-oxidative degradation of PP/GF (**Figure S6**). The evolved products from the unoxidized PP domain were investigated by analyzing the EIM curves of ion ① ($C_3H_7^+$) (**Figure S6a**). The intensities of the EIM curves for ion ① in the temperature range of 400 to 480 ˚C significantly decreased with increasing aging time. This decline can be interpreted as the oxidation of the PP matrix.

Figure S6b shows the EIM curve for ion ② ($C_6H_9^+$). The intensity of the EIM curves for ion ② in the range of 400 to 480 ˚C decreased, while the intensity of the EIM curves for $C_6H_9^+$ in the range of 200 to 400 ˚C increased with longer aging times. This trend is even more pronounced for the EIM curve of ion ③ ($C_6H_5^+$), which has a higher degree of unsaturation than ion ② (**Figure S6c**).

The evolved behaviors of oxidized hydrocarbons from the oxidized PP domain were investigated by analyzing the EIM curves of ions ④-⑥ (**Figure S6d-f**). The intensity changes for ions ④-⑥ showed a consistent increase in intensity in the 200 to 400 ˚C range with prolonged aging time. This trend was similar across all cases, despite structural differences such as oxygen content. Additionally, the simultaneous evolution of highly unsaturated products, such as ion ③, in the 200 to 400 ˚C range alongside oxidized products suggests that these unsaturated products are generated through the detachment of oxidation functional groups from the oxidized PP during the pyrolysis process.

Thus, the analysis of the temperature-dependent mass spectra revealed that unoxidized PP decomposes at higher temperatures (400 to 480 ˚C), while oxidatively degraded PP decomposes at lower temperatures (200 to 400 ˚C), corresponding to the PC-1 and PC-2 score plots, respectively.

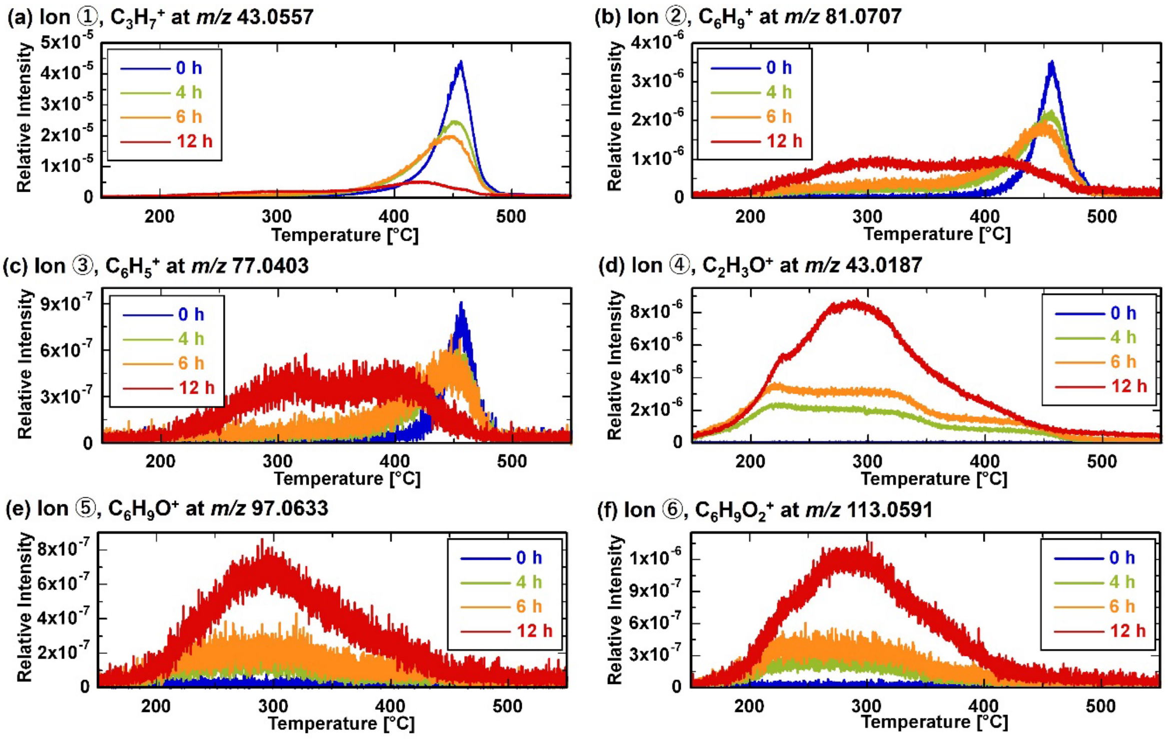

Figure S6. Evolution behavior of (a) ion ①, (b) ion ②, (c) ion ③, (d) ion ④, (e) ion ⑤, and (f) ion ⑥ from Table 1, as observed using the EIM mode of TG-TOFMS.

REFERENCES

1. Watanabe, R.; Oishi, A.; Nakamura, S.; Hagihara, H.; Shinzawa, H. Real-Time Monitoring of the Thermooxidative Degradation Behavior of Poly(Acrylonitrile-Butadiene-Styrene) Using Isothermal *In-Situ* Fourier Transform Infrared Spectroscopy Combined with Principal Component Analysis. *Polymer.* **2023**, 283. DOI: 10.1016/j.polymer.2023.126243.
2. Gugumus, F. Physico-chemical Aspects of Polyethylene Processing in an Open Mixer 2. Functional Group Formation on PE-LD Processing. *Polym. Degrad. Stab.* **2000**, 67, 35–47. DOI: 10.1016/S0141-3910(99)00115-9.
3. Socrates, G. *Infrared and Raman Characteristic Group Frequencies: Tables and Charts*, edition 3, John Wiley & Sons: New York, 2004.
4. Hu, J.; Wang, T.; Moigno, D.; Wumaier, M.; Kiefer, W.; Mao, J.; Wu, Q.; Niu, F.; Gu, Y.; Chen, Q.; et al. Fourier-Transform Raman and Infrared Spectroscopic Analysis of Dipyrrinones and Mesobilirubins. *Spectrochim. Acta A Mol. Biomol. Spectrosc.* **2001**, 57, 2737–2743. DOI: 10.1016/S1386-1425(01)00468-1.
5. Almond, J.; Sugumaar, P.; Wenzel, M. N.; Hill, G.; Wallis, C. Determination of the Carbonyl Index of Polyethylene and Polypropylene Using Specified Area under Band Methodology with ATR-FTIR Spectroscopy. *e-Polymers.* **2020**, 20, 369–381. DOI: 10.1515/epoly-2020-0041.
